# Expansion of Opportunistic Enteric Fungal Pathogens and Occurrence of Gut Inflammation in Human Liver Echinococcosis

**DOI:** 10.1128/spectrum.01453-22

**Published:** 2022-09-13

**Authors:** Yugui Wang, Aijiang Guo, Zhongli Liu, Yang Zou, Wenjun Zhu, Shile Wu, Shengying Zhang, Xiaola Guo, Shaohua Zhang, Lixia Pu, Xing-Quan Zhu, Guan Zhu, Xuepeng Cai, Shuai Wang

**Affiliations:** a State Key Laboratory of Veterinary Etiological Biology, College of Veterinary Medicine, Lanzhou University, Lanzhou Veterinary Research Institute, Chinese Academy of Agricultural Sciences, Lanzhou, Gansu, China; b College of Veterinary Medicine, Shanxi Agricultural University, Taigu, Shanxi Province, China; c Division of General surgery, Qinghai Provincial People’s Hospital, Xining, Qinghai, China; d Key Laboratory for Zoonoses Research of the Ministry of Education, Institute of Zoonosis, and College of Veterinary Medicine, Jilin University, Changchun, China; Labcorp

**Keywords:** helminths, human microbiome, opportunistic fungi, parasitology

## Abstract

Increasing evidence shows that the gut fungal mycobiota is implicated in human disease. However, its relationship with chronic helminth infections, which cause immunosuppression and affect over 1 billion people worldwide, remains unexplored. In this study, we investigated the gut mycobiome and its associations with gut homeostasis in a severe helminth disease worldwide: liver echinococcosis. Fecal samples from 63 patients and 42 healthy controls were collected to characterize the fungal signatures using ITS1 sequencing, QIIME pipeline, and machine learning analysis. The levels of fecal calprotectin and serological anti-Saccharomyces cerevisiae antibodies (ASCA) in these subjects were experimentally measured. We found that fungal microbiota was significantly skewed in disease, with an overrepresentation of Aspergillus, Candida, Geotrichum, Kazachstania, and Penicillium and a decrease of Fusarium. Machine learning analysis revealed that the altered fungal features could efficiently predict infection with high sensitivity and specificity (area under the curve [AUC] = 0.93). The dysbiosis was characterized by expansions of multiple opportunistic pathogens (Aspergillus spp. and Candida spp.). Clinical association analysis revealed that host immunity might link to the expansions of the invasive fungi. Accompanying the opportunistic pathogen expansion, the levels of fungi-associated fecal calprotectin and serological ASCA in the patients were elevated, suggesting that gut inflammation and microbiota translocation occurred in this generally assumed extraintestinal disease. This study highlights enteric fungal pathogen expansions and increased levels of markers for fungi-associated mucosal inflammation and intestinal permeability as hallmarks of liver echinococcosis.

**IMPORTANCE** Helminth infection affects over 1 billion people worldwide. However, its relationship with the gut mycobiome remains unknown. Among the most prevalent helminth diseases, human hydatid disease (echinococcosis) is highlighted as one of the most important (second/third for alveolar/cystic echinococcosis) foodborne parasitic diseases at the global level. Herein, we investigated the mycobiome and gut homeostasis (i.e., inflammation and permeability) in human echinococcosis. Our results revealed that fungal dysbiosis with an expansion of opportunistic pathogens and increased levels of fecal calprotectin and serum ASCA are hallmarks of human liver echinococcosis. Host immunity is associated with enteric fungal expansions. These findings suggest that an extraintestinal helminth infection is able to alter gut fungal microbiota and impair gut homeostasis, which resembles concomitant gut symptoms in inflammatory gut-related diseases (e.g., AIDS). In clinical practice, physicians need to take cautious medical consideration of gut health for nonintestinal helminth diseases.

## INTRODUCTION

The gut microbiome plays an important role in human health. Extensive studies to date focus on elucidating the bacterial component of the human gut microbiota in the maintenance of human health and in the progress and development of human diseases (1–3). In contrast, the fungal constituents of the microbiome (also referred to as “the gut mycobiome”) are largely understudied. However, emerging evidence indicates that gut fungi may be more important than previously thought (4), having been recognized as important players in maintaining intestinal homeostasis, regulating systemic immunity, and interacting with bacteria in ways that can be beneficial or detrimental to the host (5, 6). In particular, invasive fungal infections, which are caused by medically important fungi, including Candida spp., Aspergillus spp., Cryptococcus spp., and Pneumocystis spp., are deadly in immunocompromised conditions (7) (e.g., AIDS) and critical in the pathophysiology of some intestinal (e.g., inflammatory bowel disease [IBD]) and extraintestinal diseases (e.g., cancer and autoimmune diseases) (8, 9). Despite their importance, there is a paucity of scientific literature investigating the relationship between the gut mycobiome and the infections of parasitic pathogens.

As parasites of considerable medical and economic importance, helminths globally infect over 1 billion people and typically cause chronic infections (10). Helminths usually survive in the host over long periods of time by modulating the host Th2 immune response, which produces a profound influence on their hosts (11). In this context, helminth infection has been associated with remission of immunopathologies for immune-related diseases (e.g., allergy and IBD) or impaired immunity to coinfection with various microbial agents (e.g., bacteria and viruses), leading to increased susceptibility and attenuated immunopathology (12, 13). Furthermore, recent studies have demonstrated that the presence of helminths alters the microbiota composition and the metabolic signature of the host, and conversely, bacterial microbiota contributes to regulating host immune response to helminth or pathology (14, 15), highlighting interactions between the gut microbiome and helminth infection. However, the impacts of these diseases on the gut fungal compositions have not been established. Given that helminth infections are predominantly restricted to regions of poor sanitation where people have high exposure to microbial pathogens, including opportunistic fungal pathogens, understanding how helminth infection modulates the gut mycobiome for the host is critical.

Among the most prevalent zoonotic helminth diseases, hydatid disease (HD), including both alveolar echinococcosis (AE) and cystic echinococcosis (CE), represents a substantial disease burden, and AE and CE are respectively highlighted as the second and third most important foodborne parasitic diseases at the global level, respectively (16–18). Echinococcosis is caused by the larval stage of Echinococcus (mainly E. granulosus and E. multilocularis). Humans become infected through the ingestion of food contaminated by the worm eggs, and the liver and lungs are the most common sites of infection (19). Many of these infected people will experiencing severe clinical syndromes that are life-threatening and face reduced quality of life. AE and CE combined infect more than 1 million people worldwide at any given time, with a prevalence level as high as 5 to 10% in parts of South America, Central Asia, China, and Africa (20–22). Additionally, hundreds of millions of people are estimated to be at risk of infection in Central Asia and western China (23). However, treatment of this disease remains a challenge, and knowledge of the influence on hosts from the infection is greatly insufficient.

In this study, we performed a cross-sectional study on human liver echinococcosis to investigate the impact of this extraintestinal parasitic worm infection on the gut fungal microbiome and gut health. Our results suggest that the helminth infection greatly affected the gut fungal compositions of the host, with an expansion of opportunistic pathogens. We also found that gut fungal species could serve as noninvasive biomarkers to monitor helminth infection. In addition, biomarkers related to gut inflammation or microbial translocation were significantly increased in HD patients and were associated with the expansion of pathogenic fungi. Overall, this study highlights that helminth infection is extensively associated with alteration of the gut mycobiome and with fungi-related inflammatory features, which clinicians should pay more attention to during patient care.

## RESULTS

### Fungal microbiota is significantly skewed in hydatid disease.

We recruited 63 patients with HD (*n* = 34 for AE; *n* = 29 for CE) and 42 age-matched healthy controls after screening criteria (Table 1). To determine whether HD is associated with altering the gut fungal community, internal transcribed spacer (ITS) profiling was carried out on the 105 fecal samples collected from HD patients and healthy controls. The sequence data returned yielded 10,618,993 read pairs with an average of 101,133 reads/sample (95% confidence interval [CI], 102,962 to 99,304). After read merging and data preprocessing (i.e., filtering low quality and chimera), 63.46% of read pairs were retained (average per sample: 66,838; 95% CI, 64,640 to 63,036). After rarefaction, the sequences were clustered into 11,843 operational taxonomic units (OTUs) for further microbiome analysis. These OTUs mapped to taxa comprising of 17 phyla, 63 classes, 169 orders, 417 families, 895 genera, and 1,625 species.

**TABLE 1:** Clinical characteristics of subjects in the cross-sectional study^*a*^

Clinical indexes	Groups			*P*-values
	AE	CE	Control	
Age (yr)	33.74 ± 16.27	39.24 ± 13.09	38.38 ± 13.80	0.381
Gender (men/women)	17/17	11/18	32/10	0.001
BMI (kg/m^2^)	21.93 ± 3.55	24.47 ± 2.84	24.79 ± 4.82	0.276
Glucose (mmol/liter)	4.45 ± 0.60	4.50 ± 1.00	4.60 ± 0.41	0.198
No. of white blood cells (10^9^/liter)	6.64 ± 2.41	5.92 ± 1.63	6.28 ± 1.39	0.919
No. of neutrophils (10^9^/liter)	3.99 ± 2.43	3.53 ± 1.63	3.51 ± 1.07	0.994
No. of lymphocytes (10^9^/liter)	1.79 ± 0.62	1.86 ± 0.57	1.95 ± 0.55	0.366
No. of monocytes (10^9^/liter)	0.48 ± 0.16	0.43 ± 0.16	0.48 ± 0.15	0.426
No. of eosinophils (10^9^/liter)	0.32 ± 0.33	0.20 ± 0.22	0.29 ± 0.23	0.506
No. of basophils (10^9^/liter)	0.06 ± 0.03	0.04 ± 0.02	0.06 ± 0.02	0.172
IgG level	2.47 ± 1.42	2.63 ± 1.87	0.41 ± 0.35	<0.001

*^a^*The values are expressed in number (mean ± SD), and were compared by Mann-Whitney U test or Fisher’s exact test. BMI, body mass index.

**FIG 1:**
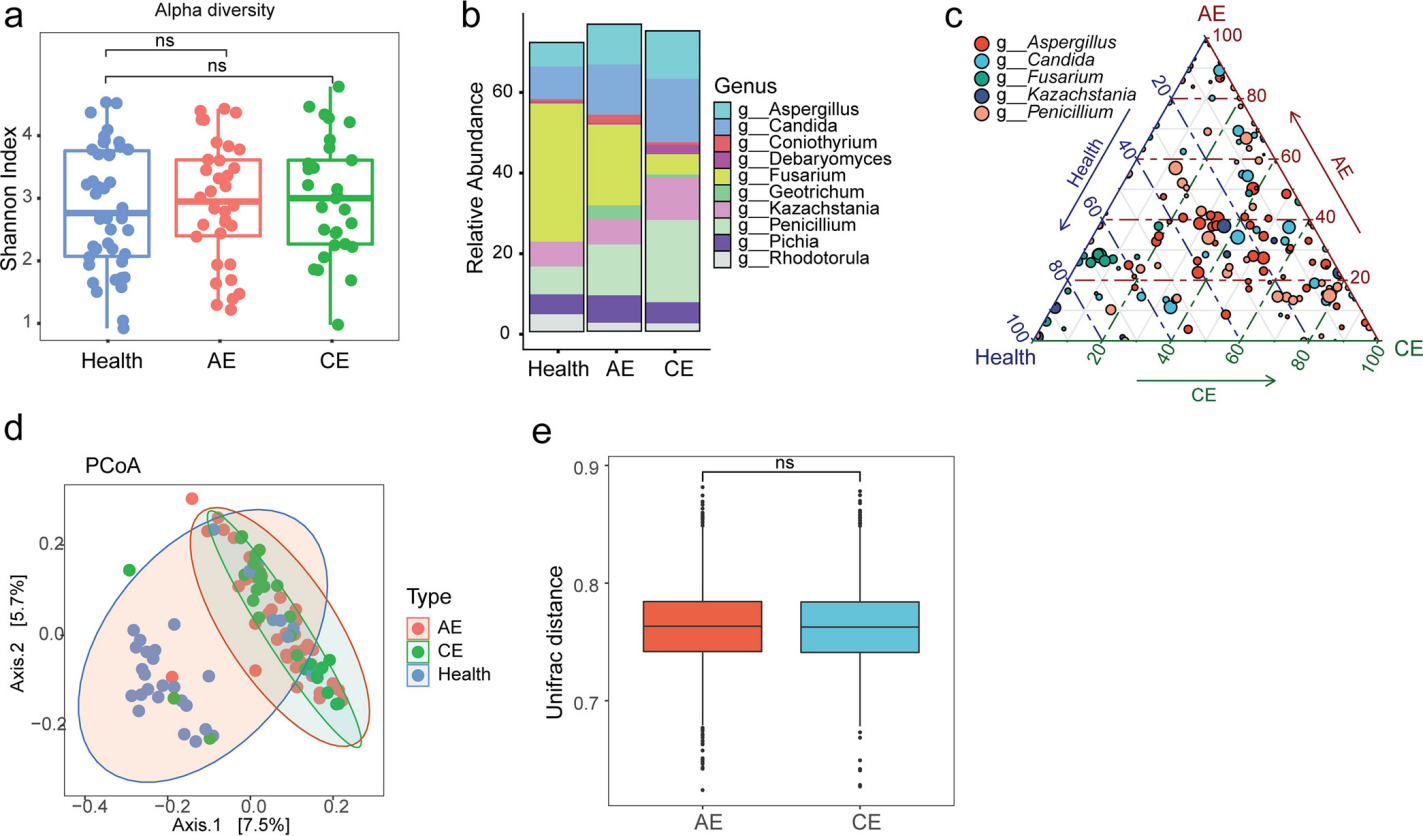
The fungal dysbiosis in hydatid disease. (a) α-Diversity as measured by the Shannon index was not significantly altered between the patients with hydatid disease (alveolar echinococcosis [AE] and cystic echinococcosis [CE]; *n* = 63) and healthy control (Health, *n* = 42). (b) The relative abundance of top 10 abundant genera in patients and healthy controls. (c) Ternary plot of phylum-level operational taxonomic units (OTUs) from the top five most abundant phyla among AE (top), healthy (left), and CE (right) subjects. Only OTUs with a taxonomy assignment are shown. The relative abundances for each OTU of the three groups in this triangular plot sum to 100%. Bubble size represents the log_2_-transformed mean abundance of each OTU. The values of each bubble (representing an OTU) on the ternary plot correspond (up to 100%) to its trilinear coordinates. Axes show reads accounted for by each OTU in each group of subjects, as a percentage of the total (sum) reads observed for a given OTU across all three groups. Arrows indicate the corresponding axis directions for each point. (d) Principal coordinate analysis (PCoA) in unweighted Unifrac distance for the groups (AE, CE, and Health). Permutational multivariate analysis of variance (PERMANOVA) results reveal that the mycobiome structure was significantly skewed between disease and healthy control (*P < *0.001) but not significantly altered between the disease forms (*P = *0.80). (e) When measured by distances to the healthy group, the AE and CE groups showed similar fungal microbial structures. ns, not significant, using the two-sided Wilcoxon rank-sum test.

We assessed and compared the species diversity in the disease and healthy cohorts. The fungal α-diversity as measured by species richness (Fig. S1a), Pielou’s evenness (Fig. S1b), and the Shannon diversity index (Fig. 1a) was not altered (*P > *0.05). In contrast, the species diversity of the mycobiome was phylogenetically elevated in the patients with HD compared to healthy controls (PD_whole tree) (Fig. S1c), indicating that OTUs in HD are phylogenetically more divergent than those in the healthy control group. At the phylum level, consistent with previous reports in healthy people (24), we found that the communities in the three groups (healthy, AE, and CE) were predominantly comprised of fungi from *Ascomycota* and *Basidiomycota* phyla, with *Ascomycota* being the most abundant phylum represented (Fig. S1d). At the genus level, the most abundant genera were skewed between HD and control. The genera, including Penicillium, Aspergillus, and Candida, were consistently overrepresented in both AE and CE (Fig. 1b). In line with this observation, we found an enrichment of OTUs from these genera in subjects with HD (Fig. 1c).

In order to further estimate whether the mycobiome was altered in the subjects with HD, we calculated the distances between the three groups (AE, CE, and healthy controls). As indicated by principal coordinate analysis with either Unifrac (Fig. 1d) or Bray-Curtis (Fig. S2) distances, the gut mycobiome discriminated HD and control into two significantly distinct groups (*P < *0.001, permutational multivariate analysis of variance [PERMANOVA]) but was distantly very similar between AE and CE (*P = *0.80, PERMANOVA). If measured by distances to control, AE and CE have similar community structures (Fig. 1e), suggesting that hydatid disease has similar effects on the gut mycobiome regardless of the disease forms. Among the investigated potential confounding factors that may have effects on the mycobiome (Table 1), only sex was found to have significant differences between the disease and control groups. A sex-matched analysis revealed the same trend of fungal alterations in this study (Fig. S3). Taken together, these data suggest that the hydatid disease was associated with alterations of the gut fungal compositions.

### Fungal features serve as biomarkers to monitor the infection of hydatid disease.

**FIG 2:**
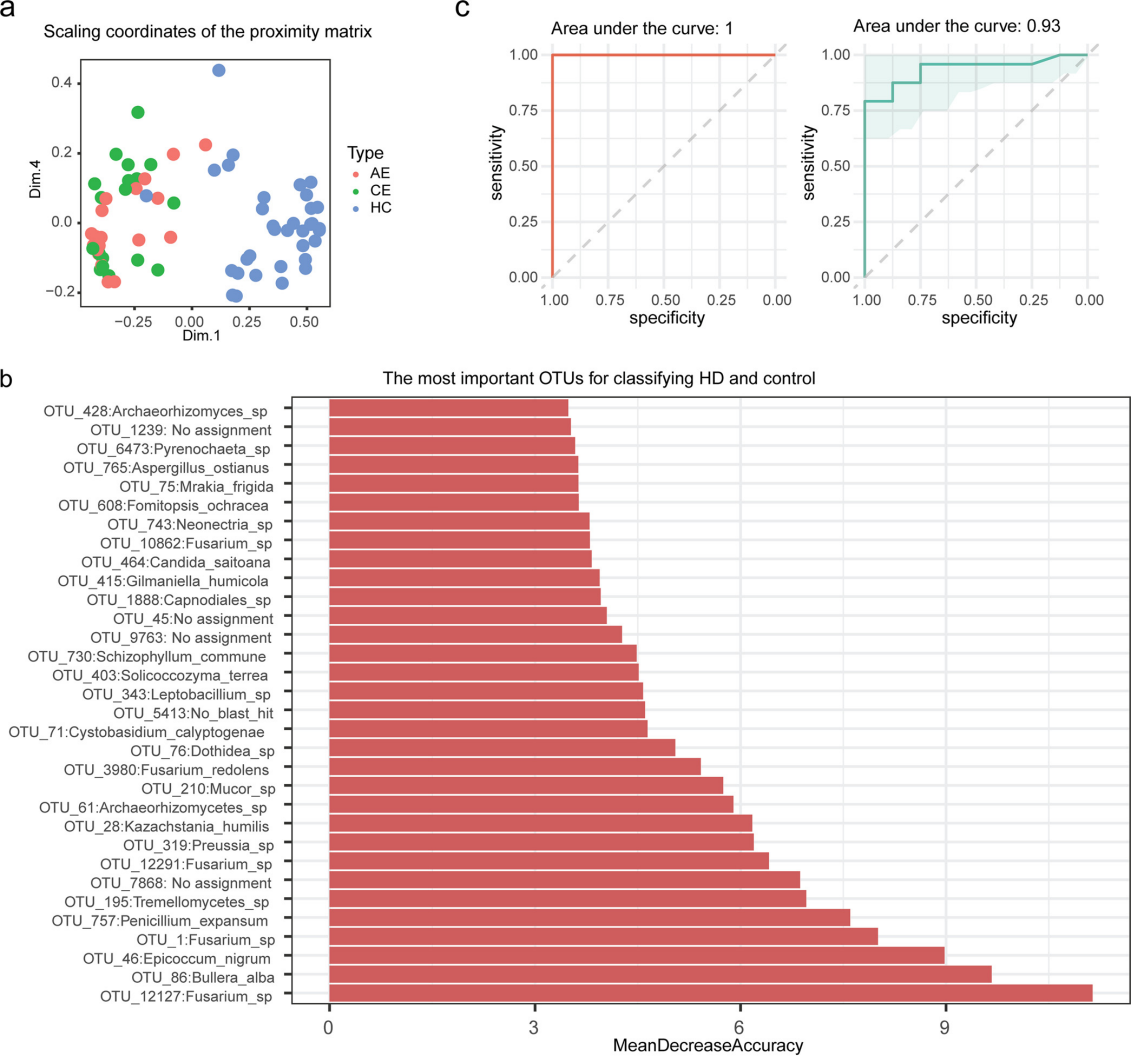
Fungal features as markers for classifying infection from control. (a) Multidimensional scaling plot of proximity matrix from randomForest for the 32 optimal markers. (b) The 32 optimal markers and their importance as indicated by mean decrease accuracy. (c) Performance of fungal markers for classification of disease from control in random forest model for training data set (left; *n* = 73) and testing data set (right; *n* = 32). The markers achieved an area under the receiver-operating characteristic curve (AUC) of 1.00 and 0.93 for the training (left) and testing (right) data sets, respectively.

Given that the gut mycobiome dysbiosis is linked to human hydatid disease, we wondered whether altered gut fungal taxa could distinguish infection from control. Using the 10-fold cross-validation of the random forest method (see Materials and Methods), we identified 32 fungal features that are significantly different in HD compared with control (Fig. 2a and b). In the cohort of HD, 11 of these OTUs were enriched, while 21 OTUs were depleted (Fig. S4). We observed that these markers classified HD and control with an area under the curve (AUC) of 1.0 in the discovery cohort (*n* = 73) for the trained random forest classifier based on these OTUs (Fig. 2c, left). The performance of this classifier identified in the discovery cohort was validated with an independent testing cohort (*n* = 32). The fungal markers distinctly stratified HD subjects from the control with an AUC of 0.93 for the testing cohort (Fig. 2c, right). Notably, the analysis using only the top 10 OTUs also provided a high accuracy for classifying groups (AUC = 1.0 for the discovery cohort and AUC = 0.91 for the testing cohort) (Fig. S5). This indicates that gut fungal markers appear to provide an efficient noninvasive approach to monitor this helminth infection.

### Opportunistic pathogenic fungi are increased in hydatid disease.

**FIG 3:**
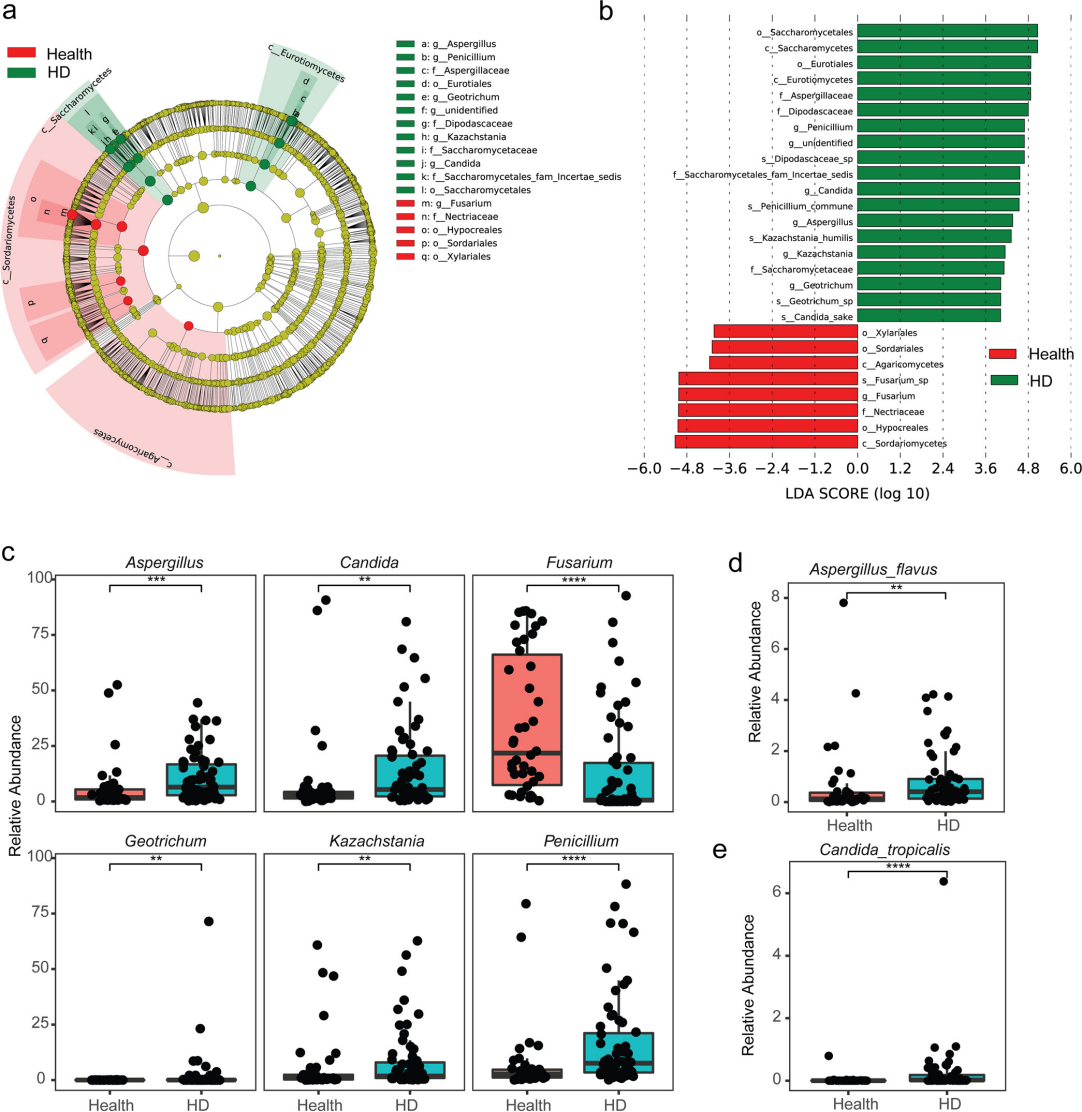
Expansions of opportunistic fungal pathogens in hydatid disease (HD). (a) Cladogram of the taxa with differential abundance in LDA effect size (LEfSe) analysis. The maximum number of taxonomic levels is six for plotting. (b) The effect size of each differentially abundant feature is estimated by linear discriminant analysis (LDA) in Lefse. Only features with a logarithmic LDA score of >4 and a false discovery rate (FDR) of <0.05 are shown. (c) Relative abundances of the genera enriched in differential abundance analysis. (d, e) Relative abundance of Aspergillus flavus and Candida tropicalis between HD and healthy control. **, *P < *0.01; ***, *P < *0.001; ****, *P < *0.0001 using the two-sided Wilcoxon rank-sum test for panels c to e.

The above evidence of dysbiosis promoted us to carry out a formal differential abundance analysis using LDA effect size (LEfSe) to identify the enriched taxa in HD, compared with control. Apparent alterations of fungal compositions at different levels were observed between HD and healthy control (Table S1), including an expansion of classes *Euotiomycetes* and *Saccharomycetes* (yeast) and depletion of classes *Agaricomycetes* and *Sordariomycetes* in the HD group, compared with control (Fig. 3a and b). For the most differentially abundant taxa at the genus level, Penicillium, Aspergillus, Candida, Kazachstania, and Geotrichum were significantly more prevalent in the patients with hydatid disease, whereas Fusarium was decreased in these patients (Fig. 3b and c).

Notably, opportunistic pathogens from Candida and Aspergillus were abundant in the gut mycobiome of HD (Table S1). In the elevated taxa in HD (logarithmic linear discriminant analysis [LDA] score > 2 and α-value < 0.05), species from Aspergillus (e.g., Aspergillus infrequens, Aspergillus rugulosus, Aspergillus versicolor, and Aspergillus flavus) and species from Candida (including Candida sake, Candida solani, Candida parapsilosis, and Candida tropicalis) are well known as the most common opportunistic human pathogens, especially in immunocompromised individuals (Fig. S6 and S7). For example, A. flavus (Fig. 3d), and C. tropicalis (Fig. 3e), which were among the most frequent species involved in individuals with AIDS, were significantly increased in HD. Some other well known causative agents for fungal infections, such as Trichosporon asahii (a species that can cause severe opportunistic infections [trichosporonosis]), Wallemia sebi, and Talaromyces rugulosus, were also increased in the patients with HD (Table S1). Collectively, these results suggest that the increase of opportunistic pathogenic fungi in the gut is a signature of hydatid disease.

### Expansion of the opportunistic pathogenic fungi is associated with host immunity.

**FIG 4:**
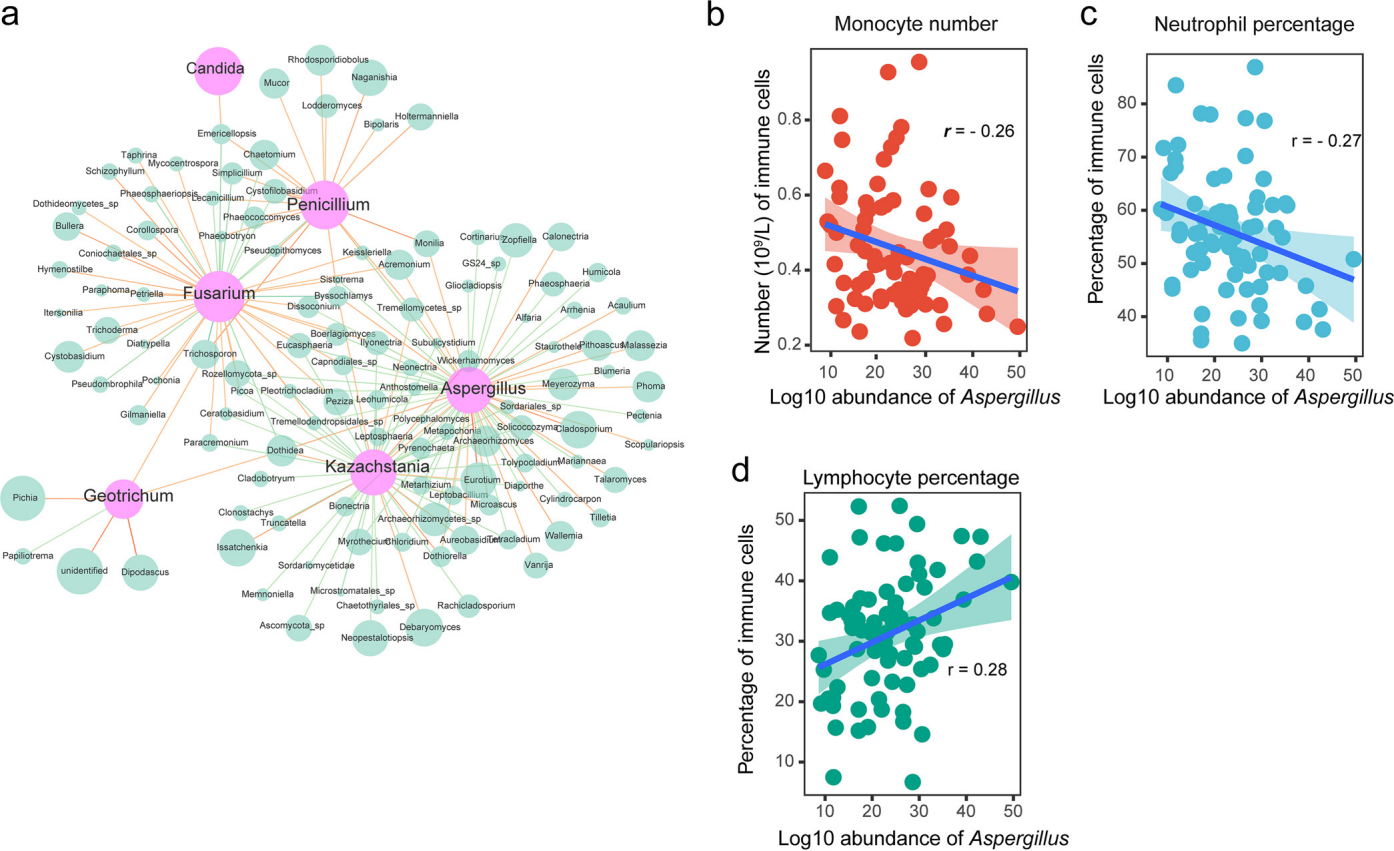
Variables associated with opportunistic fungal pathogens. (a) Correlation network between taxa at the genus level. Positive correlations (orange) and negative correlations (green) are shown as line links (FDR < 0.05, absolute value of correlation [*r*] > 0.3). The node size represents logarithmic mean abundance for all the samples. (b to d) Spearman correlation (*r*) between Aspergillus and immune cells for the patients. The linear model is shown with a 95% confidence interval. The false discovery ratio was used for correcting *P* values of multiple hypothesis tests.

Next, we tried to dissect what factors are linked to the alterations of the fungal community regarding the helminth infection. We first accessed the potential interactions between the altered taxa and others. Network analyses revealed that most of these fungi were extensively linked with other compositions (Fig. 4a; false discovery rate [FDR] < 0.05, absolute *r *> 0.3). Aspergillus had the densest linkage, whereas Candida had the fewest number, for which only one positive correlation with Emericellopsis was measured. These results suggest that the wide interactions between compositions might be associated with the expansion of the opportunistic pathogenic fungi.

We next investigated whether the fungal dysbiosis was associated with the host immune. We analyzed the routine blood indices of the HD patients and controls. The genera Aspergillus, Penicillium, and Geotrichum were significantly correlated with immune cell levels (monocytes, neutrophils, and lymphocytes) in the blood (FDR < 0.05) (Table S2). Interestingly, Aspergillus (also Penicillium) is negatively correlated with monocyte number or neutrophil percentage but is positively correlated with the percentage of lymphocytes (Fig. 4b to d) (FDR < 0.05), suggesting that host immunity is another factor associated with the changes in the gut mycobiome.

### Fungi-related gut inflammation and microbial translocation occurred in hydatid disease.

**FIG 5:**
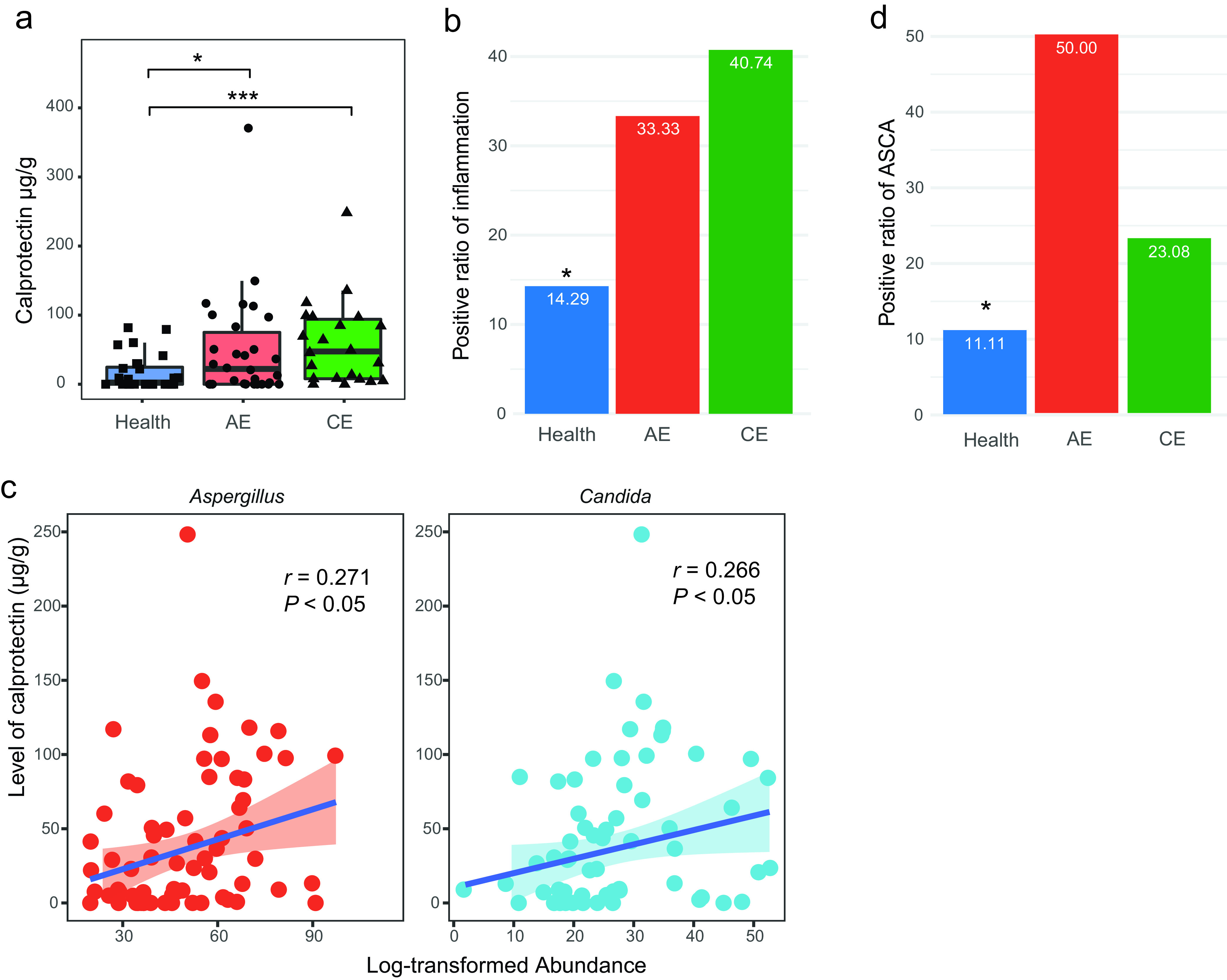
Inflammation and microbial translocation markers increased in hydatid disease. (a) Levels of calprotectin in feces of patients and healthy controls. (b) Positive ratio of inflammation, as indicated by a cutoff of 50 μg/g calprotectin in feces. (c) Correlation between levels of calprotectin and abundance of either Aspergillus or Candida for the patients. Spearman correlation (*r*) was calculated for all these results. (d) Positive ratio of anti-Saccharomyces cerevisiae antibodies (ASCA) in serum. *, *P < *0.05; ***, *P < *0.001 using two-sided Wilcoxon rank-sum test for panel a, and Fisher’s test with an odds ratio for panels b and d.

There is evidence that the gut barrier is damaged in patients with extraintestinal diseases (e.g., HIV and autoimmune hepatitis). The overrepresentation of pathogenic fungi in the patients with HD and their associations with host immunity prompted us to test whether changes in intestinal inflammation status or gut permeability occurred in HD. We assessed the fecal calprotectin concentration, which is the most reliable and routinely used marker for gut inflammation of IBD (also is elevated in HIV) (25), in the patients and the healthy controls. Median calprotectin concentrations were markedly increased in the patients with HD (HD: 53.22 μg/g, 95% CI, 33.98 to 72.46 μg/g; AE: 49.1 μg/g, 95% CI, 20.80 to 77.31 μg/g; and CE: 58.90 μg/g, 95% CI, 32.20 to 85.60 μg/g), in comparison with that in control (14.24 μg/g, 95% CI, 5.42 to 20.06 μg/g) (Fig. 5a) (*P* < 0.01). If a calprotectin level of less than 50 μg/g is considered negative for suggesting gastrointestinal inflammation (which is generally used in clinical diagnosis for IBD), the positive ratio of calprotectin in fecal samples was significantly increased in HD (33.33% for AE and 40.74% for CE) in comparison to that in control (14.29%) (*P < *0.05, odds ratio = 3.48, 95% CI, 0.99 to 15.79) (Fig. 5b). For the patients with HD, a significantly positive correlation was observed between the relative abundance at the genus level of either Aspergillus (*r* = 0.271, *P < *0.05) or Candida (*r* = 0.266, *P < *0.05) (Fig. 5c) and the level of fecal calprotectin.

As gastrointestinal diseases are usually linked with microbiota translocation characterized by the presence of fungi antigen antibodies in serum, we estimated the intestinal permeability by examining the prevalence of anti-Saccharomyces cerevisiae antibodies (ASCA) in serum of patients with HD and healthy controls. The prevalence of ASCA, which has cross-reactions with other yeast (e.g., Candida spp.) (26), was detected in 34.78% of patients with HD (50.00% of patients with AE and 23.08% of patients with CE), while only 8.89% of healthy controls were ASCA positive (*P < *0.01, odds ratio = 5.37, 95% CI, 1.53 to 24.32) (Fig. 5d), indicative of potential fungal translocation in the gut of HD patients. Collectively, these data indicate that human hydatid disease is characterized by increased levels of markers for microbial translocation and gut inflammation.

## DISCUSSION

Helminths cause chronic infections of over 1 billion people around the world (11), creating a widespread acquired immunocompromised condition. Recent evidence from human and animal studies indicates that helminth infections, in particular of intestinal helminth, can influence the gut bacterial microbiota (27). In contrast, few studies have established a link between helminth infection and fungal compositions. In this study, we investigated the effect of the extraintestinal helminth infection (“hydatid disease”) on the gut mycobiome of patients. The results revealed for the first time that helminth infection is associated with a marked fungal dysbiosis in the gut. This disease-specific dysbiosis is apparently featured by the expansion of some well known opportunistic pathogens, for example, A. flavus, and C. tropicalis, strongly suggesting a detrimental impact from the fungal shift. This is in accordance with the observation that the markers for inflammation or microbiota translocation were significantly increased in the gut of patients with HD in this study. For the clinical practice of nonintestinal helminth (e.g., larval forms of Taenia, Fasciola, and Trichinella) diagnoses or treatments, patients’ gastrointestinal health or fungal infection is not routinely examined. The findings in this study suggest that chronic nonintestinal helminth infection is able to induce unexpected influences on the intestine, which commonly happens with immunodeficiency diseases (e.g., HIV) (28, 29). As a result, physicians may need to carefully gain medical awareness of the intestinal health of patients with helminth infection, at least for human hydatid disease; restoring gut microbial homeostasis by antifungals during or after treatment may also be essential for patient care.

The most significant finding in this study is that the increase of pathogenic fungi was associated with HD. Species from yeast Candida spp. and mold Aspergillus spp. represent the most medically important fungi and are responsible for various invasive human diseases (7, 30). It is not clear how these pathogens expanded in abundance in the gut of the patients with HD and whether these expansions could cause mucosal infection needs further validation. Increased Candida at the genus level in several liver diseases (31, 32) and IBD (32–34) have been reported previously, suggesting that Candida species are likely associated with pathologies of host liver or intestinal diseases. Notably, the most common fungal infections of the gastrointestinal tract in patients with IBD were caused by Candida species (32, 33, 35). For Aspergillus, increased levels were also observed in ulcerative colitis (36) and liver diseases (37). Our results of the taxa interaction network and correlation analysis with routine blood tests suggest that the mechanism underlying the overgrowth of Candida may be distinct from that for Aspergillus, as Aspergillus is more likely to link with host immune responses and has more interactions with other taxa. Nevertheless, both Candida and Aspergillus species are the fungal pathogens that frequently cause invasive fungal disease in immunocompromised persons overall (7, 28), suggesting that these opportunistic pathogens were potentially linked to the immunocompromised situation caused by HD infection. Indeed, the clinical data in this study further pointed out that host immunity, which is generally characterized by immunosuppression with high-level interleukin 10 (IL-10) expression and Treg cell expansion, as well as T-cell exhaustion during hydatid disease (38), links to the fungal expansions. In line with this notion, Penicillium, which is commonly found in HIV patients (28), was also significantly overrepresented in this study. Although it remains undetermined whether these pathogenic fungi expansions could cause harmful or or even life-threatening systemic intestinal infections under the situation with HD, increasing cases of concomitant pulmonary or liver echinococcosis and aspergillosis have recently been reported (39–42). As a result, preoperative verification of the presence of local aspergillosis might be practically necessary. However, this study used ITS1 sequencing for profiling the fungal mycobiome. As a limitation of this study, the fungal compositions observed in this study might represent only portions of the whole fungal community. Future investigations based on untargeted sequencing or culture-based methods are needed for further confirmation.

Hydatid disease might be associated with the disruption of gut homeostasis for the patients, as suggested by the biomarkers of inflammation and microbial translocations. Fecal calprotectin concentration, which relates directly to the severity of the inflammation, is not only a robust determinant of the activity degree for IBD (43) but also favors monitoring gut status for numerous gut-related diseases, such as hepatocellular carcinoma (44), cirrhosis (45), and AIDS (25). In this study, we observed a significant increase of this protein in the feces of the patients for either AE or CE, suggesting that gut inflammations occurred during the infection. This is an interesting observation, as extensive previous studies have associated various helminth infections with a reduced degree of autoimmune diseases. The yeast or mold in the gut may link to the inflammation of the gut, as a positive correlation was found between Candida/Aspergillus and the concentration of calprotectin in our results. The potential of microbial translocation in the gut of patients with HD support this notion and further implies that the gut barrier for individuals with HD may have been weakened, enabling fungi (e.g., yeast) in the gut to enter the bloodstream. Leaky gut syndrome is associated with many gut-related diseases, like IBD, HIV, and other autoimmune diseases (lupus, type 1 diabetes, and multiple sclerosis) (46). In this regard, leaky gut syndrome has been rarely recorded in helminth infection, while preservation or perturbation of gut barrier function has been reported for circumstances with different intestinal helminth infections (47). In this study, the observation that higher serum ASCA levels were found in the patients with HD suggests an increase in gut permeability. A “leaky gut,” with an increased translocation of S. cerevisiae (or related antigens), could thus be one potential explanation for the increased ASCA IgG concentrations. However, elevated serum levels of anti-S. cerevisiae antibodies do not exclusively result primarily from a defect of the gut barrier, as many healthy subjects in this study also show a relatively high level of this antibody, which has also been found in other studies (48). Whether the increased level of ASCA in HD is associated with leaky gut needs further pathological evidence. It is possible that altered epithelial permeability could be due to the host immune response to fungi or helminth-derived factors (e.g., ES products). Additionally, the degree of gut barrier dysfunction may be related to disease forms of HD, as AE and CE showed different degrees of ASCA levels, but the roles of mycobiome at this point are totally undefined. Further studies need to elucidate these questions, as they affect how to restore gut homeostasis for these patients.

The results of this study also revealed fungi can potentially serve as an accurate and noninvasive surrogate marker of hydatid disease. We identified 32 OTUs that could characterize the disease by the random forest model trained based on the cohorts in this study. These taxa can effectively and accurately distinguish the samples with or without infection, indicative of the potential of the fungi-based diagnosis method for nonintestinal parasite infections. Together with the results of fecal calprotectin, this evidence supports the notion that bioinformation from feces is useful in monitoring the disease activity and guiding treatment in the clinical setting of parasitic diseases. The clinical diagnosis of HD is most set by ultrasound or other imaging techniques such as computed tomography (CT) scan, combined with epidemiologic history. As a high proportion of infections are usually asymptomatic, the diagnosis of liver hydatid cyst may often be incidental. A high percentage of patients with HD gets their diagnosis incidentally when they are seeking medical care for other reasons (49). Thus, a large-scale preliminary screening would highly benefit the population with a history of infection exposure in areas of endemicity. Enzyme-linked immunosorbent assay (ELISA) may be a feasible candidate method for such a mission. However, serology tests for HD diagnosis usually suffer from false-positive reactions, loss of detectable antibodies (seronegative), and low compliance with blood sampling procedures. In this regard, a noninvasive approach based on a set of fecal microbiome biomarkers, which has been proposed using machine learning in this study, might be a useful alternative for large-scale HD screenings. In addition, this method would be a good addition to the clinical definitive diagnosis of this disease, which routinely requires a combination of imaging, serologic, and immunologic studies. Importantly, our results showed that a set of 10 OTUs was sufficient to distinguish infection from control with high accuracy, suggesting that the development of a method (e.g., quantitative PCR [qPCR]) more feasible than the targeted sequencing would be possible. Further studies to investigate the robustness of these markers and their clinical significance in HD are warranted. Additionally, longitudinal studies are needed to elucidate the dynamics of fungi compositions during infection so as to identify disease status-related biomarkers.

In conclusion, this study highlights the importance of the gut mycobiome in helminth infection and reveals that fungal dysbiosis and loss of homeostasis might be hallmarks for patients with nonintestinal helminth infection. Although little attention has been paid to intestinal homeostasis during the clinical treatments of various helminth infections, in particular in the cases of nonintestinal parasites, our results suggest that chronic helminth infection was associated with unexpected influences on the intestine. Thus, physicians may need to cautiously consider intestinal health in patients with helminth infection during clinical practice.

## MATERIALS AND METHODS

### Patient cohorts and sample collection.

In this cross-sectional study, participants of 63 patients with hydatid disease and 42 healthy controls were recruited at Qinghai Provincial People’s Hospital, Xining, China. The consolidated standards of reporting trials (CONSORT) diagram for this study is shown in Supplemental File 1. Individuals with hydatid disease enrolled in this study are all Tibetans who live similar lifestyles (nomadic), have similar diets (animal-based diet), and are inpatients before any medication for liver echinococcosis. The diagnosis of liver echinococcosis by physicians at the division of general surgery followed criteria based on HD-specific IgG ELISA, ultrasonographic features, CT scan, and pathological check after surgery (Supplemental File 2). Briefly, a history of epidemiology, clinical manifestations, and IgG ELISA were used for preliminary diagnosis of the disease, and ultrasonography was used for confirmation and clinical differentiation of liver echinococcosis (AE or CE). If the ELISA was positive or a liver lesion appeared on the ultrasound, a CT scan was employed to provide a comprehensive display of the characteristics of hydatid lesions and confirmation of disease type (AE or CE). The differentiation between AE and CE was mainly based on the imaging examinations of ultrasonography and CT, as described previously (50). A final pathological check of the lesion after surgery was performed to further confirm the diagnosis results. Healthy controls were age-matched volunteers without any medications from the physical examination center in the hospital, who are also Tibetans from the same community as the HD patients and also live a nomadic lifestyle with an animal-based diet. The diagnosis of HD for healthy controls followed the same criteria for HD patients except for CT and pathology checks after surgery. Suspected cases with any liver lesions were excluded from the control group. Exclusion criteria for both the disease and control groups were as follows: participants with any chronic diseases (such as cancers and diabetes), nonviral hepatic diseases (such as autoimmune hepatitis, liver cirrhosis, alcoholic liver disease and nonalcoholic fatty liver disease), chronic viral diseases (such as HIV, HBV and HCV), or on either antibiotics or antifungal medications within the last 3 months were excluded. The data of routine blood tests were retrieved from the diagnosis step at admission. Stool samples for each participant during the initial diagnostic procedure for screening on the day of admission before any treatment were collected and divided into aliquots of 200 mg and stored at −80°C until used.

### ITS sequencing and data processing.

Fecal sample DNA was extracted using fecal DNA extraction kit (magnetic soil and stool DNA kit, Tiangen) according to the manufacturer’s instructions. The DNA samples were used for ITS1 gene sequencing for fungi (51, 52). Briefly, the ITS1-5F region was amplified using the primers ITS5-1737F (5′-GGAAGTAAAAGTCGTAACAAGG-3′) and ITS2-2043R (5′-GCTGCGTTCTTCATCGATGC-3′) with barcodes. The DNA libraries were constructed by TruSeq DNA PCR-free sample preparation kit (Illumina) following the manufacturer’s recommendations. The library quality was assessed on the Qubit 2.0 fluorometer (Thermo Scientific) and Bioanalyzer 2100 system (Agilent) and sequenced by NovaSeq6000 (Illumina). Raw paired-end reads (250 bp) were filtered with barcodes and merged into raw tags by FLASH (version 1.2.7) (53). Quality control of the raw tags was performed using Qiime1 pipeline (version 1.9.1) (54) with default settings, and chimeras were removed by Vsearch (version 2.18) (54, 55). The clean data were clustered into operational taxonomic units (OTUs) at 97% identity level by Uparse (version 7.0.1001) (56). The representative sequence for each OTU was subject to taxonomy annotations by searching against the curated Unite database (version 8.2) (57) using the blast-based pipeline in Qiime 1 software package (version 1.9.1). As a comparison, the denoising method of data set 2 (58) implemented in QIIME2 (59) was used to group similar sequences of the clean data together based on amplicon sequence variants (ASVs), and the classify-sklearn method was used to assign taxonomy for ASVs. The main results from QIIME2 are shown in Fig. S8.

### Data analysis.

Singletons were removed, and the OTU table was rarefied to 46,474 sequences per sample. The R package Phyloseq (60) was used for analyses of α diversity and beta diversity. Differential analysis was performed by LDA effect size (LEfSe) (logarithmic LDA score of >2 and α-value of <0.05) implemented in Galaxy (version 1.0) (https://huttenhower.sph.harvard.edu/galaxy/) and by the analysis of compositions of microbiomes (ANCOM) method in ANCOMBC package (61) (zero_cut = 0.8). The results of ANCOM are shown in Table S3 for comparison. Spearman correlation was computed between the abundance of each fungal composition at different levels (log-transformed) or between the abundance of fungal taxa and the value of each variable (i.e., routine blood indices and concentrations of calprotectin in feces). To avoid taking log of the zero value, we added one read to the abundance for each composition before calculation. For cooccurrence analysis, fungal microbial taxa must make up a maximum relative abundance of 0.001% across the samples and present in >10% of all samples, and the links between taxa (*r* > 0.3 and FDR < 0.05) were analyzed in Cytoscape (version 3.8.2) (62).

Random forest regression models were constructed with the rarefied OTU count data. OTUs present in <1% of all samples were filtered out for further analysis. The samples used for fungal composition profiling in this study were randomly partitioned into a training data set and a validation data set with a proportion of 7:3 for both the patient and healthy control cohorts. The optimal set of fungal markers for the prediction of disease activity of the training data set was chosen by 10-fold cross-validation (replicates = 5) of a random forest model (The R package randomForest 4.6–14) with default parameters (“importance = TRUE”). The point with the minimum cross-validation error was viewed as the cutoff point, and the first 32 features were selected as intestinal markers based on the ranked value of MeanDecreaseGini. The identified optimal set of top 32 features was applied to test the capability of prediction for the training data and validation data in the final “bagged” random forest classifier as evaluated by the receiver operating characteristic (ROC) curve (pROC package). The area under the ROC curve (AUC) was used to designate the ROC effect. Multidimensional scaling of proximity matrix from randomForest for the 32 optimal markers was plotted by MDSPlot (R package randomForest 4.6–14).

### Measurement of fecal calprotectin and serum ASCA.

Calprotectin levels in feces were detected by ELISA according to the manufacturer’s protocol (Legend max human MRP8/14 ELISA kit, Biolegend). In brief, 50 mg frozen feces were dissolved in 1 mL PBS for 30 min and vortexed for 5 min. The homogenized samples were centrifuged with 3,000 × *g* for 5 min at 4°C, followed by 10,000 × *g* for 10 min at 4°C. The supernatant was removed and stored at −80°C until used. Calprotectin concentration was determined at an optical density at 450 nm, and the results were determined from the standard curve, including a total sample dilution of 1:2,500. Serum samples for ASCA analysis were collected from an independent cohort of patients with HD and healthy controls following the same screening criteria. The serum samples were collected and stored at −80°C until used. Levels of ASCA in serum (*n* = 20 for AE, *n* = 26 for CE, and *n* = 45 for healthy control) were quantified by enzyme immunoassay following the manufacturer’s protocol (ASCA IgG ELISA kit, Abnova). The level of ASCA (U/mL) was measured at 450-nm wavelength, and a sample with an ASCA level higher than 20 U/mL was defined as positive.

### Statistical analysis.

Statistical analyses were performed using R (version 4.1.0). The abundance of taxa, α-diversity, and distance between samples were compared using the Wilcoxon rank-sum test (two-sided; confidence level of 0.95) between groups. β-Diversity was measured using permutational multivariate analysis of variance (PERANOVA) with the Adonis function from the vegan package (version 2.4-0). Fisher’s test (two-sided; confidence level of 0.95) and odds ratios were used to compare the positive ratios between different groups for either calprotectin concentrations or ASCA levels. The *P* values for the multiple hypothesis tests were corrected by false discovery ratio (FDR). Values of *P < *0.05 or FDR < 0.05 were considered significant.

### Ethics.

This cross-sectional study has been approved by the Clinical Research Ethics Committee of Qinghai Provincial People’s Hospital (reference 2021-161). Written informed consent was obtained from each participant.

### Data availability.

The raw sequence data are available in the Sequence Read Archive (SRA) of the U.S. National Center for Biotechnology Information (NCBI) under BioProject PRJNA778607. All data relevant to the study are included in the article or uploaded as online supplemental information.
