# Supplementary material for: Expansion of Opportunistic Enteric Fungal Pathogens and Occurrence of Gut Inflammation in Human Liver Echinococcosis
Source: Microbiol Spectr. 2022 Sep 13;10(5):e01453-22. doi: 10.1128/spectrum.01453-22 (PMC9602787; doi:10.1128/spectrum.01453-22)
Supplement: Supplemental file 1 — Supplemental material. Download spectrum.01453-22-s0002.pdf, PDF file, 2.9 MB [file spectrum.01453-22-s0002.pdf]

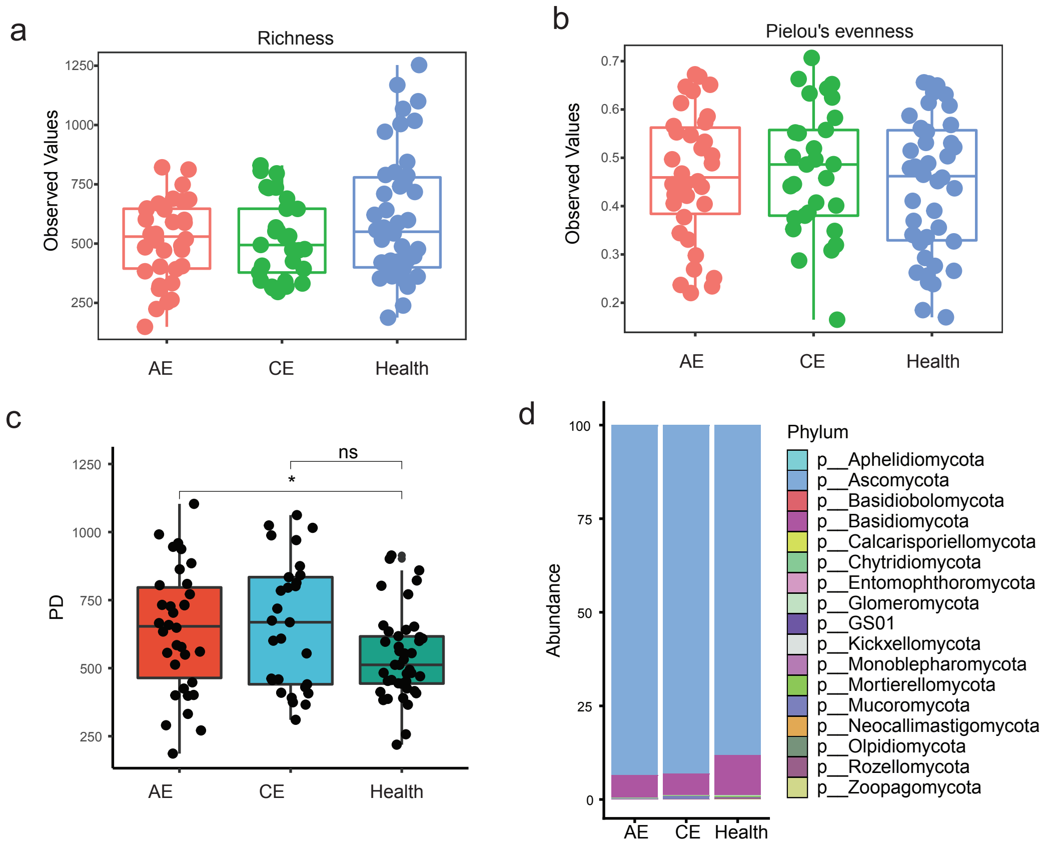

Fig. S1. The fungal community in hydatid disease and control. The richness (a) Pielou's evenness (b), and phylogenetic distance (c) of fungal compositions are shown for the three groups. The community structure at the phylum level (d) is shown with relative abundance. **P* < 0.05, using two-sided Wilcoxon rank-sum test.

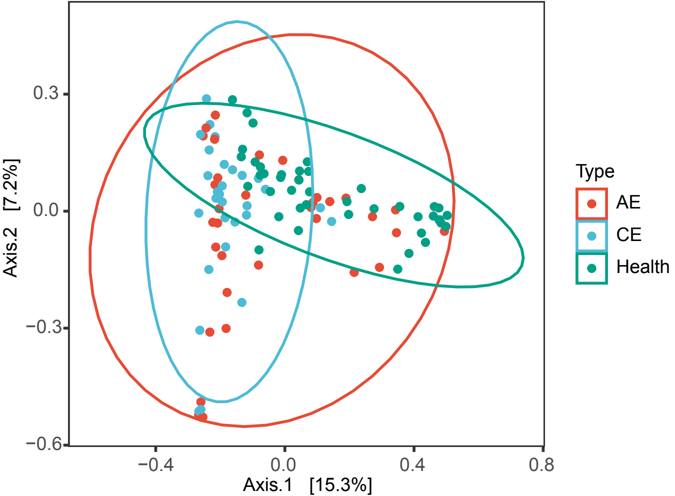

Fig. S2. Principle Coordinate analysis (PCoA) based on Bray-Curtis distance.

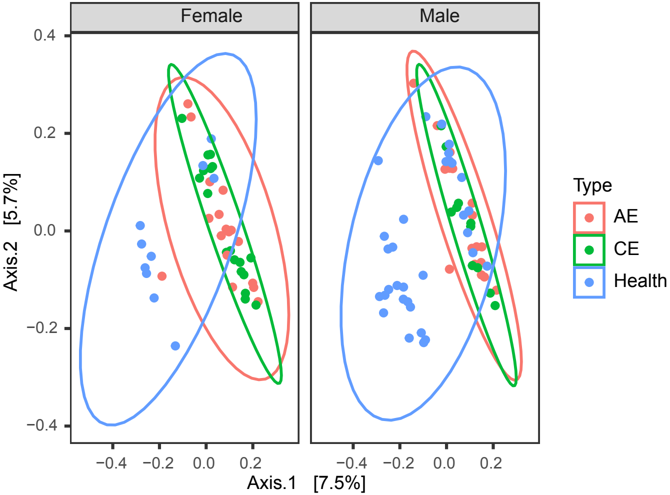

Fig. S3. Sex-matched Principle Coordinate Analysis (PCoA). The distances of Unweighted Unifrac were calculated for PCoA.

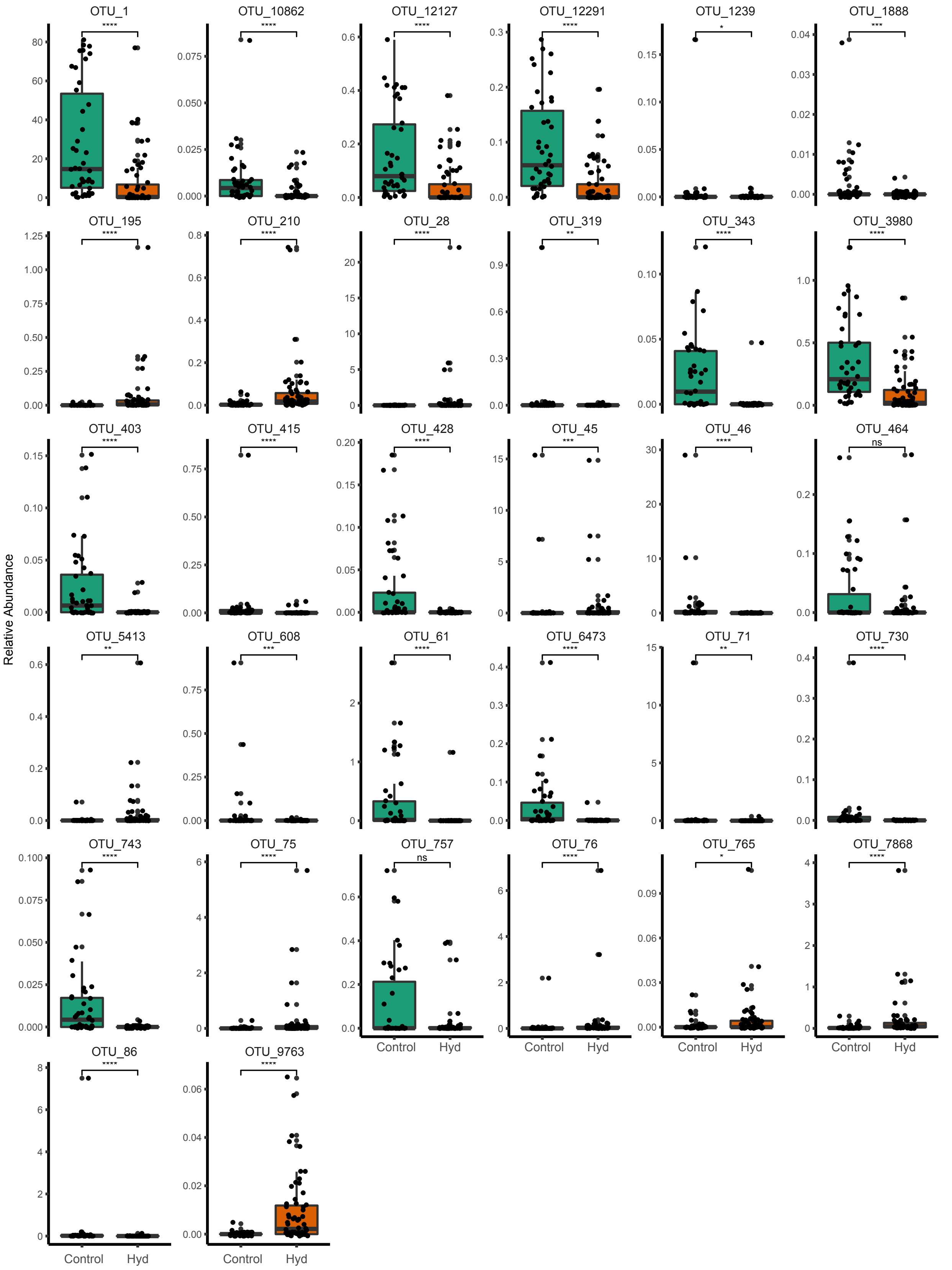

Fig. S4. Fungal OTUs as markers for classifying infection from control. The 32 optimal markers with relative abundance in hydatid disease (hyd) and control are shown. ns, not significant, ***FDR* < 0.01, ****FDR* < 0.001, *****FDR* < 0.0001 using the two-sided Wilcoxon rank-sum test.

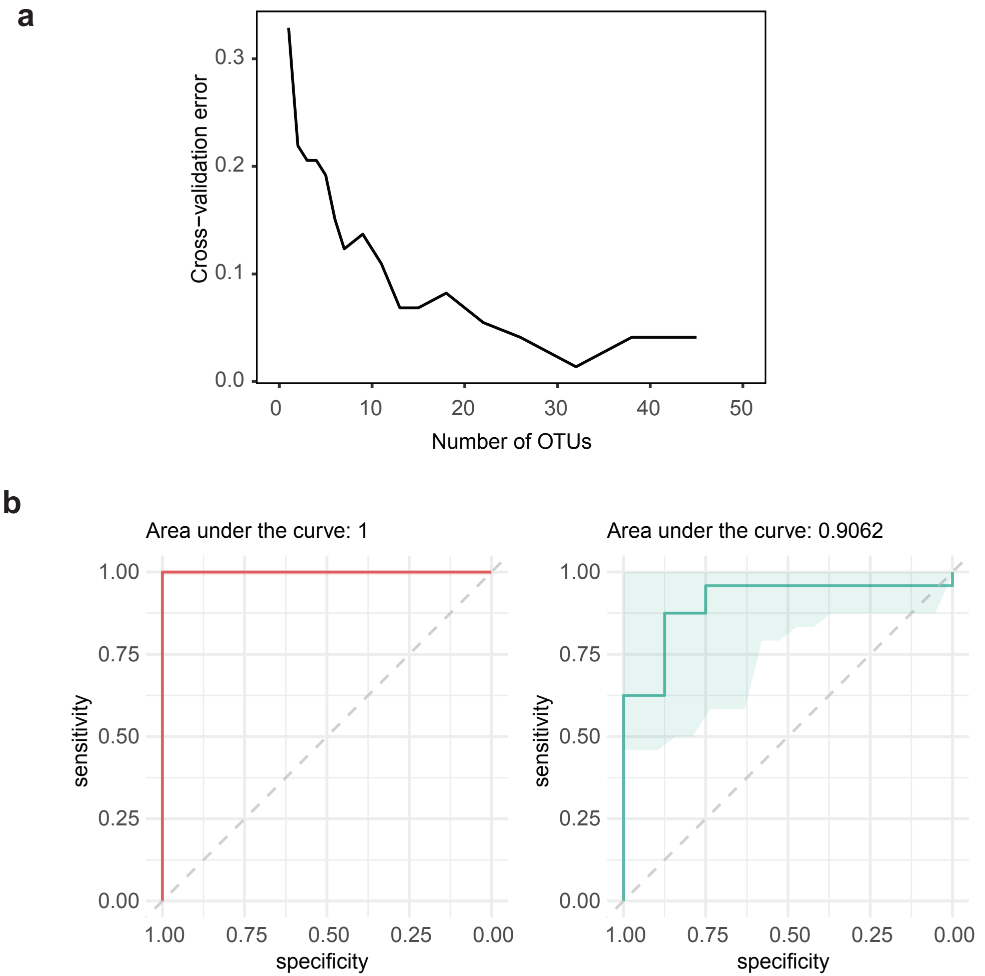

Fig. S5. The top 10 OTU markers for classifying infection form control. The cross-validation errors are shown for top OUTs that are ranked by their importance in the random forest model (a). The top 10 markers achieved an area under the receiver-operating characteristic curve (AUC) of 1.00 and 0.91 for the training (left) and testing (right) datasets, respectively (b).

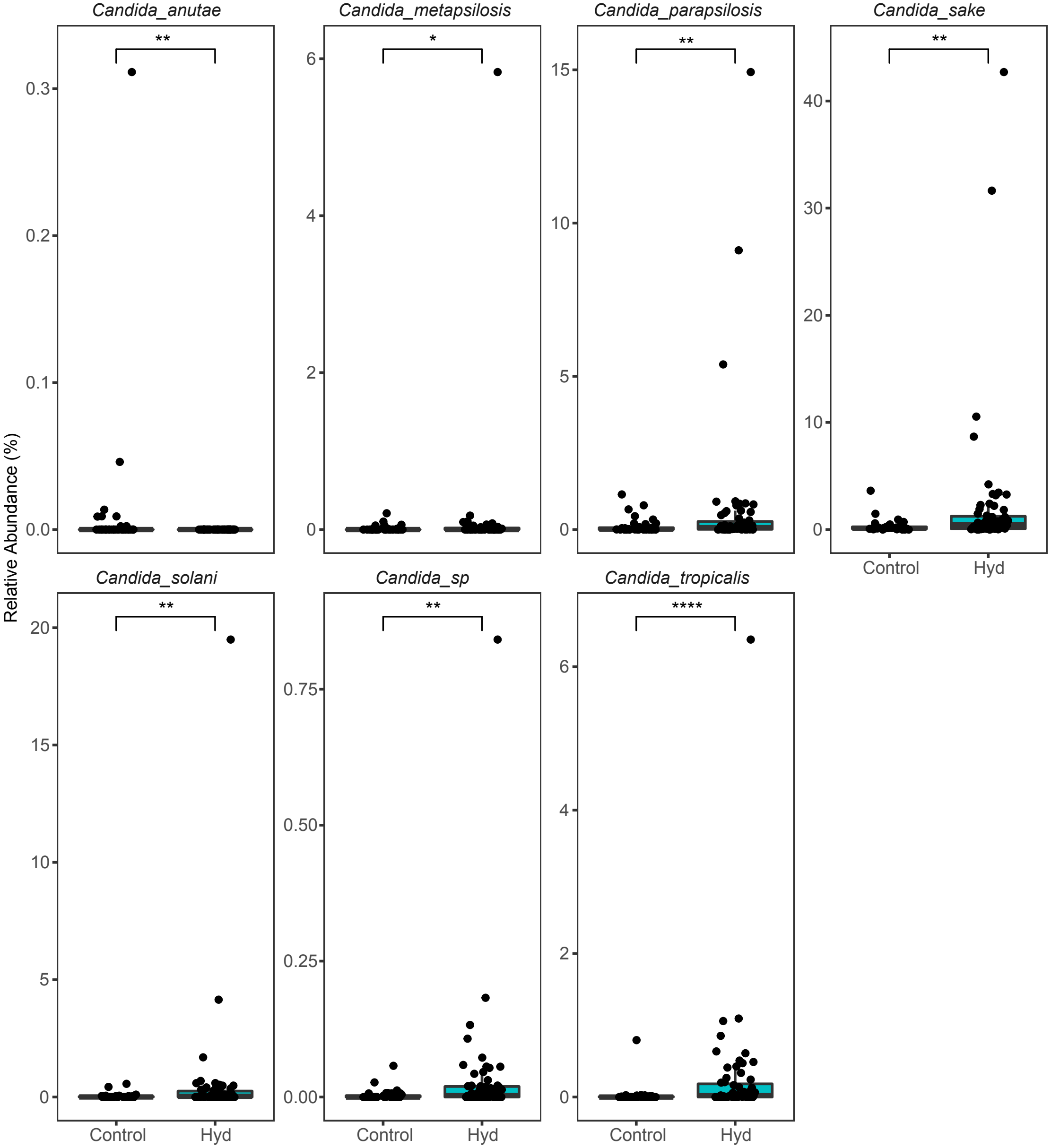

Fig. S6. The Candida species with differential abundances in healthy control (control) and hydatid disease (Hyd). Lefse was used to identify species with differential abundance in the two groups (logarithmic LDA score > 2 and Alpha-value < 0.05). **FDR* < 0.05, ***FDR* < 0.01, ****FDR*< 0.001, *****FDR*< 0.0001 using the two-sided Wilcoxon rank-sum test.

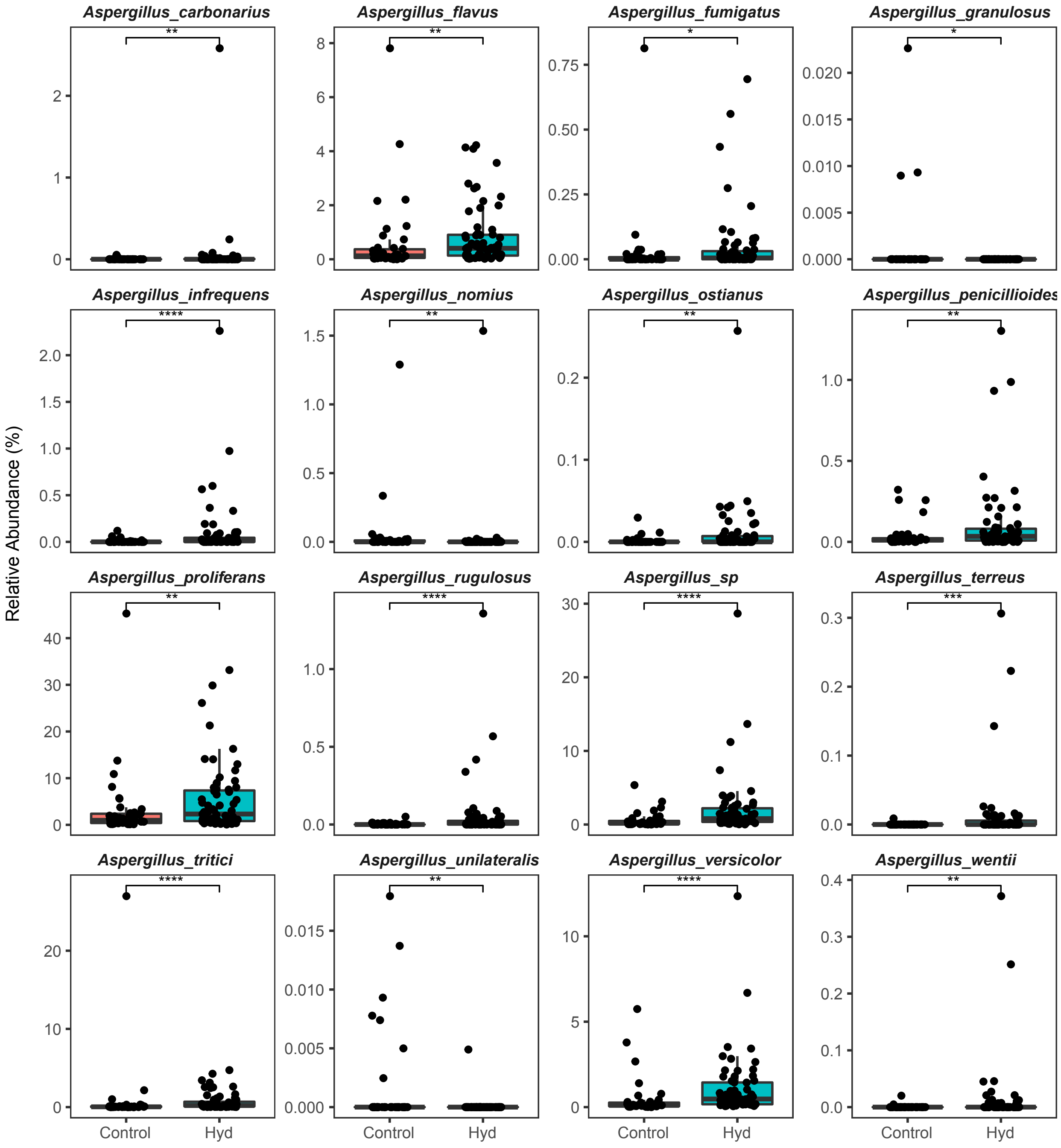

Fig. S7. The Aspergillus species with differential abundances in healthy control (control) and hydatid disease (Hyd). Lefse was used to identify species with differential abundance in the two groups (logarithmic LDA score > 2 and Alpha-value < 0.05). **FDR* < 0.05, ***FDR* < 0.01, ****FDR* < 0.001, *****FDR* < 0.0001 using the two-sided Wilcoxon rank-sum test.

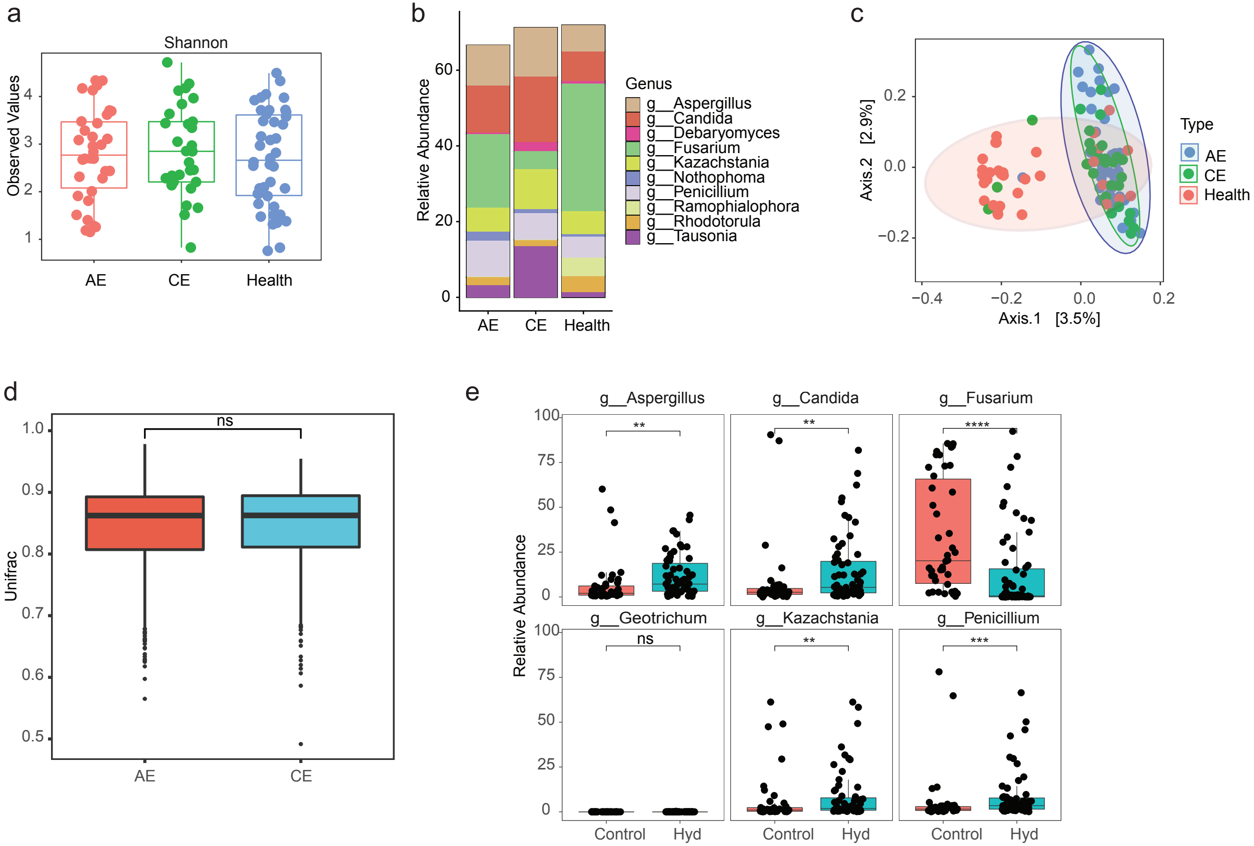

Fig. S8. The results for the QIIME2 pipeline. Shannon index for alpha diversity (a), relative abundances for top 10 abundant genera (b), PCoA for the groups (c), Unifrac distance between AE and CE (d), and relative abundances of the genera enriched in differential abundance analysis (e) are shown. $^{**}P < 0.01$, $^{***}P < 0.001$, $^{****}P < 0.0001$ using the two-sided Wilcoxon rank-sum test for panel e.

Supplementary File 1. The CONSORT diagram for this cross-sectional study.

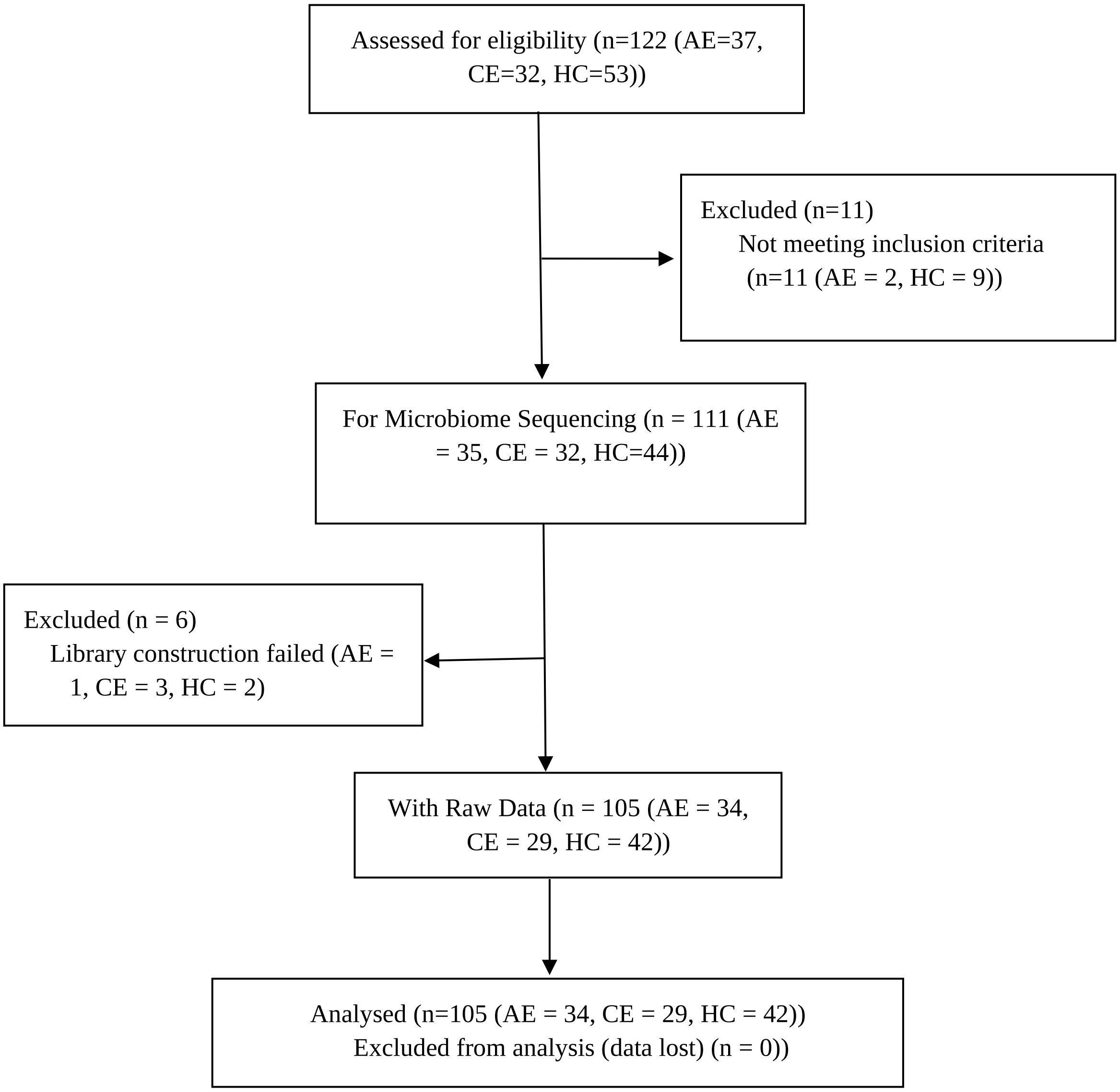

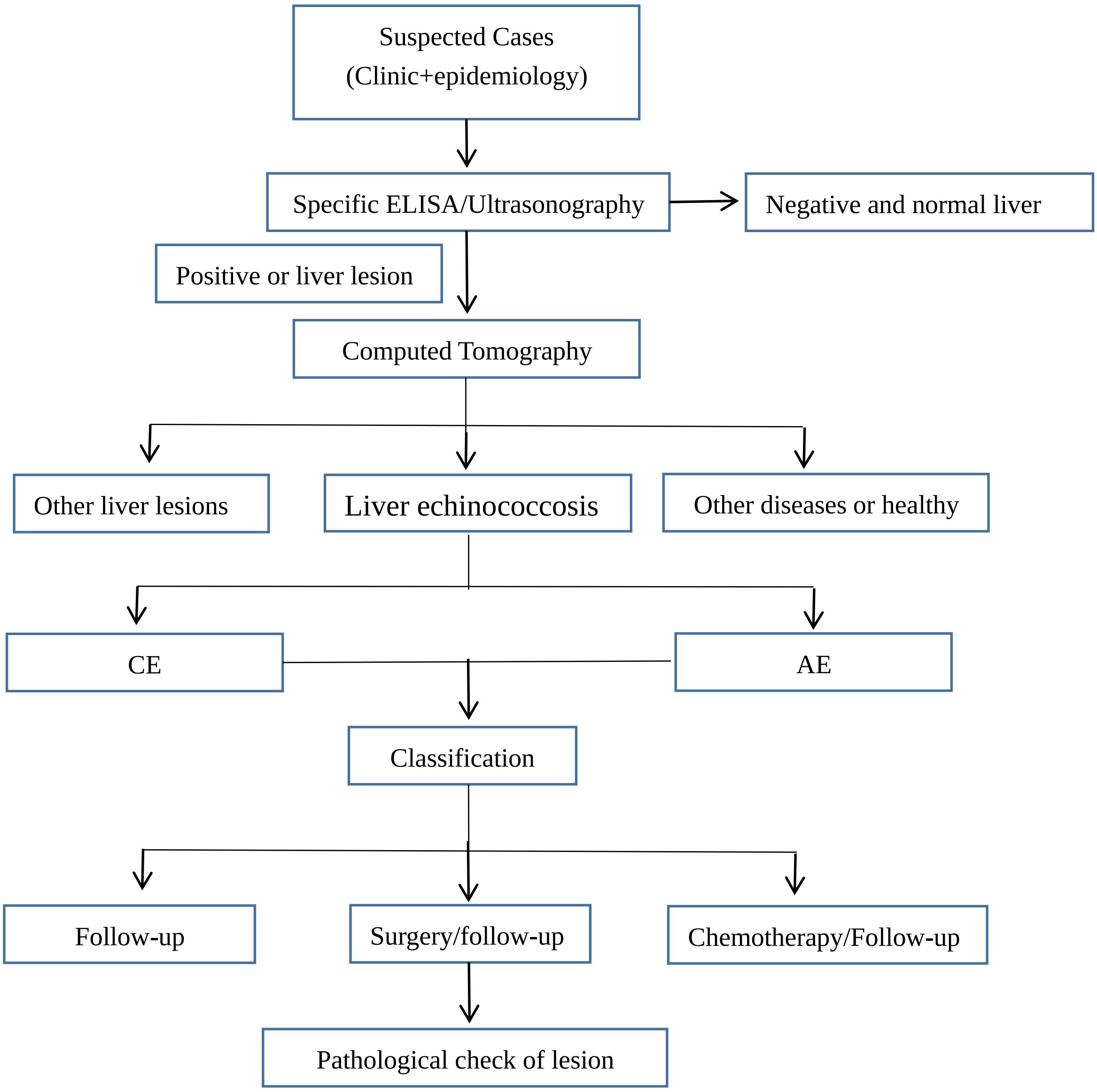

Supplementary File 2. Diagnosis process for hepatic hydatid disease. History of epidemiology, clinical manifestations, and IgG Elisa were used for preliminary diagnosis of the disease, and Doppler ultrasonography was used for confirmation and clinical differentiation of liver echinococcosis (AE or CE). If the ELISA test is positive or liver lesion was found, Computed Tomography scan was employed to provide a comprehensive display of the characteristics of hydatid lesions and confirmation of disease type (AE or CE). A final pathological check of the lesion after surgery was performed to further confirm the diagnosis results. A follow-up examination in 3-6 months was recommended.
